# Love Island, Social Media, and Sousveillance: New Pathways of Challenging Realism in Reality TV

**DOI:** 10.3389/fsoc.2019.00059

**Published:** 2019-08-02

**Authors:** Xavier L'Hoiry

**Affiliations:** Department of Sociological Studies, University of Sheffield, Sheffield, United Kingdom

**Keywords:** Love Island, sousveillance, social media, surveillance, reality TV, audience participation

## Abstract

This paper explores the changing nature of audience participation and active viewership in the context of Reality TV. Thanks to the ongoing rise of social media, fans of popular entertainment programmes continue to be engaged in new and innovative ways across a number of platforms as part of an ever-expanding interactive economy. *Love Island* 2018 has pushed the boundaries of this participatory culture by exploiting new forms of digital media in order to encourage multi-platform consumption of content by the show's fans. This paper argues that while this strategy has enabled *Love Island* to successfully exploit monetization opportunities, it has simultaneously created opportunities for the show's audience to group together online and form communities of resistance which have placed themselves in opposition to the show's producers. These fan communities have harnessed the connective powers of social media to pool together their means and knowledge and to eventually exercise modes of sousveillance designed to hold “powerful” actors to account for perceived wrongdoing on the show. Examples of such behavior during *Love Island* 2018 hint at a paradigm shift in the relationship between television producers and audiences and demonstrate the new pathways available to audiences as they seek to answer the perennial question of this entertainment genre: how real is Reality TV?

## Introduction

In the past two decades, considerable scholarly analysis has reviewed the growth and proliferation of different forms of Reality TV (Nabi, 2007; Andrejevic, 2008). With most commentators agreeing that Reality TV has “moved from the margins of television culture to its core” (Orbe, 2008, p. 345), a central theme of academic discussion has been the extent to which this form of entertainment truly offers realism and authenticity (Biressi and Nunn, 2005; Hall, 2009; Hill, 2014). Reality TV's claims to assert a “radical inclusiveness and transparency” (Kjus, 2009: 281) is a key appeal to audiences (Papacharissi and Mendelson, 2007), but the issue of realism continues to dominate discussions and the question—for audiences and scholars—often remains: how real is Reality TV (Escoffery, 2006)? Relatedly, academic discussions of Reality TV have also focused on the ways in which this televisual format has sought to generate audience participation. This, it is argued, has led to the transformation of viewers from passive consumers to active participants as part of an ever-expanding interactive economy (Andrejevic, 2004; Holmes, 2004). This paper argues that the latter of these developments has recently begun to impact the former—audiences engaged within a participatory culture are increasingly querying the authenticity of Reality TV. As will be illustrated below, this process has been aided by the rapid expansion of social media in the past 15 years and the connective power of platforms such as Twitter. So while social media provides television producers with considerable potential for multi-platform exposure of their content as well as new pathways toward engagement with audiences, these same platforms also empower individuals by bringing them together in more cohesive fan communities who are able to share knowledge and pool resources (Lévy, 1997). As a result, what appears to have emerged are increasingly “savvy” (Andrejevic, 2008, p. 27) fan communities who, encouraged to proactively participate with their favorite television shows and supplied with the tools to do so via social media, pursue behaviors perhaps unforeseen, unexpected and ultimately damaging to these very same shows as they challenge Reality TV's claims to realism and authenticity. *Love Island* 2018, in which the show's fan community mobilized online to exercise modes of surveillance—or more accurately sousveillance (Mann et al., 2003)—as part of their interaction and engagement with the show, serves as a particularly salient example.

The paper begins by briefly tracing the historical development of attempts to elicit viewer engagement across different forms of popular entertainment, culminating at the turn of the twenty-first century with the introduction of Reality TV shows such as *Big Brother*. Next, the paper briefly discusses notions of authenticity and realism in the context of Reality TV and audience expectations. The paper then considers how a contemporary form of Reality TV, *Love Island*, has pushed the boundaries of audience participation by exploiting the connective potential of social media, and in doing so, how *Love Island's* producers have reaped commercial rewards. The discussion then outlines concepts from surveillance scholarly literature such as lateral surveillance and sousveillance to explain the participatory nature of these forms of watching and monitoring which have grown considerably in recent years thanks to social media. The final section of the paper brings these strands together and draws upon examples from *Love Island* 2018 to demonstrate how audiences have re-imagined their engagement and participation and re-purposed this to exercise modes of sousveillance to hold the show's producers to account.

## Reality TV and the Interactive Economy

Contemporary audiences of Reality TV are expected and prompted not to be passive consumers but active participants shaping the day-to-day narrative and ultimately co-producing outcomes (Holmes, 2004). In some ways, these forms of audience engagement are not new and in fact have a long tradition preceding the advent of Reality TV. Kjus (2009) outlines the way classic American radio-based games and quizzes such as *Vox Pop, Idol*, and the *Major Bowes Amateur Hour* provided early examples of entertainment as a “combination of social engagement and responsibility” for audiences (2009, p. 279). Such programming encouraged “ordinary” listeners to take part in the production of content and the opportunity to determine outcomes. These forms of social engagement and participation were later followed by television-based programmes, most notably *The §64,000 Question*, a quiz format subsequently re-developed, re-hashed, and re-booted time and time again across many different countries over the next several decades. The next iteration of this growing participatory culture came in the creation of popular day-time talk shows such as *Oprah*, in which “ordinary” people were once again tasked with generating content, this time in an increasingly politicized context as audience members were recruited to discuss pressing social issues and contribute to debates. Again, the success of this format prompted many similar versions to emerge in the US and beyond, replicating the format in which a participatory platform fronted by a pseudo-political host encouraging audience members to contribute to debate, initially in person in the studio but, as time went on, via telephone, text, email, and of course in recent years via social media (Kjus, 2009).

Despite these televisual formats offering pathways in which the public could participate in these programmes, the nature of this participation was heavily regulated, and the agency of audience members and viewers at home remained limited. For one thing, individuals selected to participate were often carefully chosen and their image curated to exploit particular characteristics or to chime or clash with the sensibilities of home audiences (Anderson, 1978). But what also marks out audience participation in these contexts as compared to that which Reality TV would later claim to offer is that participants engaging in quizzes and talk shows were only expected to either answer set questions or offer opinions on pre-determined topics. Moreover, viewers at home were largely still consuming these shows passively since their ability to contribute was also restricted in the same ways. As such, audiences were still pre-dominantly passive in the sense that they had few opportunities to determine outcomes or genuinely contribute to the development of narratives.

The introduction of Reality TV shows at the turn of the twenty-first century such as *Big Brother* and *Pop Idol* was the beginning of a rapid paradigm shift which began to restructure the “interface between industry, text and audience” (Holmes, 2004, p. 214). While popular docu-soaps such *The Real World, Cops*, and *The Osbournes* certainly contributed to the advancement of Reality TV during the 1990s (Doyle, 1998; Gillan, 2004), these formats still cast the viewer as passive recipients. *Big Brother* on the other hand, heralded the move toward expressly, and deliberately empowering viewers to shape outcomes by enabling them to choose, week by week, who remained in the show, thus co-opting the audience to co-produce what happened next. Kjus describes this key development as “a shift from the asymmetrical communication of broadcasting to the symmetry of telephony and the Internet” (2009, p. 295). As Holmes (2004) has noted, this apparent empowerment of audiences was not merely a sideshow within these programmes but rather it was placed at the heart of their design and marketing. This was perhaps most obviously demonstrated in the slogans used to promote these shows which emphasized the central role of audiences and their empowerment–“*You* decide!” (Big Brother) and ‘But this time *you* choose!’ (Pop Idol) (Holmes, 2004).

This ability to co-produce outcomes and determine directions of the narrative may be thought of as the democratization of production within an increasingly interactive economy (Andrejevic, 2004). This growing interactivity was facilitated by technological advancements in the early twenty-first century, most obviously the move toward Web 2.0 representing a shift from static webpages to dynamic and collaboratively constructed online content, including the rise of social media platforms (O'Reilly, 2005). The true extent of this interactivity has been queried by some and Jenkins (2006, p. 3) in particular has argued that despite constantly new and emerging forms of audience engagement in television and other media, “not all participants are created equal… and some consumers have greater abilities to participate in this emerging culture than others.” Whether this participatory culture truly encompasses and embraces all audiences is perhaps unclear, but what is certain is that this move toward an interactive economy in television consumption was ultimately designed for the benefit of television producers and their sponsors first and foremost. This shift toward interactivity was necessary if television as medium was to keep up with its competition. In the mid-1990s, commentators such as Negroponte (1995, p. 54) predicted a dire future for “passive old media” such as television broadcasting due to the impending explosion of “interactive new media” facilitated by the internet. Indeed, Gilder (1994, p. 189) rejected the idea that television could continue to exist alongside new media, claiming that “the computer industry is converging with the television industry in the same sense that the automobile converged with the horse.” Although these warnings ultimately proved to be rather over-stated, recent developments in media production arguably revisit these concerns and may even have made them ever-more pressing. Entertainment industry giants such as Netflix and Amazon (and soon Apple) now have not only the platforms to deliver content via their streaming websites but also the resources necessary to commission, produce, and market their content unilaterally. Competition for audiences has perhaps never been more fierce and this has therefore arguably pushed television producers to seek new and innovative ways to keep their audiences engaged.

Moreover, audience participation can, of course, be monetized. These monetization processes take place overtly—in the first incarnations of *Big Brother*, for instance, telephoning (and eventually texting) to vote for your favorite housemate would be charged. But monetization can also be rather more subtle thanks to the opportunities to generate advertising revenues within these shows, a process described by Deery(2004: 1) as “advertainment” in which “shows themselves act as marketing vehicles in addition to attracting audiences for spot advertisers.” In the contemporary digital era, opportunities for advertainment have grown exponentially, thanks in part to the creation of mobile apps and the use of these apps as the exclusive medium through which audiences can participate in the co-production of shows' outcomes. Once they have downloaded a show's official app, users are soon confronted with a panoply of marketing for the show in question as well as its many commercial partners. But as Razaghpanah et al. (2018) have explained, mobile apps are also armed with the capacity to collect users' data, revealing their consumer preferences and habits, enabling more targeted advertising and, ultimately, greater potential for monetization. Though writing in the mid-2000s, Jenkins predicted the potential for greater commercial exploitation mediated through a merging of Reality TV and digital media. He proposed that when television begins converging with other forms of media such as the internet, “every important story gets told, every brand gets sold, and every consumer gets courted across multiple media platforms” (2006, p. 3).

This redefinition of traditional relationships and passive/active dichotomies is arguably best exemplified in a relatively recent example of Reality TV—*Love Island*. In particular, the extensive and deliberate use of social media made by producers and audiences has pushed audience participation in new and perhaps unexpected directions.

## Reality TV, Authenticity and Realism

Notions of realism and authenticity are at the heart of ongoing debates concerning Reality TV. Academic work has often discussed authenticity in Reality TV by examining the extent to which depictions of certain populations can be said to be authentic and representative of reality (Escoffery, 2006). But Hill (2014) has argued that rather than making claims to absolute authenticity, Reality TV overtly invites audiences to explore the fluid nature of realism, performance and identity. Jones (2003) has further argued that audiences are in fact aware that Reality TV is far from authentic but deliberately suspend disbelief in order to indulge in something of a “guilty pleasure.” Similarly,Allen and Mendick(2013, p. 466) propose that rather than seeking a complete sense of realism, audiences in fact derive enjoyment from trying to distinguish the real from the false in Reality TV shows and that this “ambiguity provides space for pleasure.” This may explain the considerable popularity of shows such as *The Hills, Keeping up with the Kardashians* and many others which are billed as Reality TV despite widespread acknowledgment that scenes are scripted and key events carefully choreographed (Woodward, 2018). However, others have argued that the promise of realism in Reality TV continues to represent a key appeal for audiences. Papacharissi and Mendelson (Papacharissi and Mendelson, 2007, p. 363) research has shown that for audiences, “the more realistic reality TV programming was perceived to be, the greater the affinity viewers experienced, and vice versa.” In a rather more abstract sense, Fetveit (1999, p. 798) has argued that Reality TV offers a symbolic connection to realism for its audiences and that “a powerful urge for a sense of contact with the real is inscribed in much of the reality TV footage.” Further, Hill (2002, p. 324) has claimed that a perennial attraction for audiences of Reality TV is the potential to capture a “moment of authenticity” amongst contestants, as exemplified by the recurring use of devices such as “reveals” and “confessionals” in these shows.

These scholarly discussions are particularly pressing when considered against the long history of subterfuge in Reality TV programming. For instance, Anderson (1978, p. 14) has explored quiz show scandals in the US in the 1950s, as part of which producers supplied personable and well-liked contestants with answers in order to keep them on the air for as long as possible. Meanwhile, “hard cases, whiners, or smart alecks” were systematically filtered out of broadcasts (1978, p. 14) and quizzes ostensibly depicted as fairly rewarding the most intelligent contestants were shown to be doing anything but. In the UK, the mid-2000s saw its own Reality TV scandal when widespread fraudulent activities were uncovered concerning audience participation in quizzes and contests. This form of participation was found to have been subject to abuse and manipulation including inventing winners of prizes; faking the results of contests which had charged audiences for taking part; failing to count telephone and text votes due to technical errors; overcharging individual callers on premium telephone lines; and broadly misleading viewers as to the nature of the games and quizzes they were taking part in. The sheer scale of the scandal and the financial cost to viewers was unprecedented and labeled as the “the biggest fraud in UK TV history” (Deans, 2007). The aftermath of this scandal included record fines against broadcasters, high-profile resignations and calls for legal amendments to protect audiences from similar abuses in the future (Deans, 2007).

It may thus be argued that the legacy of such incidents is a lasting sense of broken trust amongst Reality TV audiences and an entirely justified skepticism as to claims of authenticity. Heritage (2019) has proposed that this shattered trust endures today and is exemplified by constant accusations of manipulation which emerge during broadcasts of major Reality TV programmes in the UK. However, Heritage also argues that rather than justifying claims of “fixes” in Reality TV, the scandal of 2007 has in truth led to much stricter regulation of such programming. Nevertheless, audiences' trust has been irrevocably damaged, and this paper will argue that this continuing distrust can be demonstrated by the activities of contemporary audiences in their ongoing search of authenticity. When audiences sense they have been duped, they react pro-actively and–thanks to social media–collectively.

## Love Island, Social Media, Audience Participation, and Monetisation

The scholarly work discussed above enables an appreciation and understanding of the role of Reality TV as a conduit for reinventing and re-envisioning the extent to which audiences can be engaged in co-production of content. But what these analyses perhaps lack is a recognition of the impact social media in particular would come to have upon the nature and extent of this participatory culture. *Love Island's* embracing of social media hints at the vast potential to further the increasingly symbiotic relationship between television and digital media as simultaneous sites of consumption. *Love Island's* merging of television broadcasting with social media demands an acknowledgment that audiences must be re-configured as occupying dual roles—that of television viewers *and* social media users. Moreover, *Love Island* also presents a case study in the potential for exploitation of new and emerging forms of media consumption and the opportunities for monetization this offers. Equally however (as will be argued later), *Love Island* may also demonstrate the unexpected consequences that may arise when “viewers-users” increasingly embrace this duality and all the potential it may offer.

*Love Island* is a British Reality TV dating show during which contestants spend 8 weeks in a villa in Spain. Contestants are tasked with “coupling up,” meaning they must find a partner and avoid being “single” and consequently being removed from the show. Single contestants are removed on a weekly basis following a so-called “re-coupling” ceremony during which contestants decide who they wish to “couple up” with. During the course of the show, contestants go on dates, take part in challenges and broadly interact in the villa under the constant gaze of a production crew filming their activities. Over the show's 8 week run, the *Love Island* audience are invited to take part in voting on a number of topics, some critical to the show's narrative and others rather more mundane. Examples include (but are not limited to) voting for: favorite/least favorite couple; which contestants should go on a date; which contestants should leave the show; and which contestants should receive various forms of preferential treatment. At the end of the show, the audience is ultimately tasked with voting for the winning couple from those to have made it to the final episode. Reality TV shows centered on the premise of dating and romance have a long history and indeed represent one of the most prolific genres of Reality TV in the past two decades. Shows such as *The Bachelor, Beauty and the Geek*, and *Millionaire Matchmaker* have achieved global success while others such as *Dinner Date* and First *Dates* have dedicated followings in the UK and abroad (Campelli, 2015). What all of these shows lack however is any conduit for participation and audiences are instead rigidly cast as viewers passively consuming content. *Love Island* has therefore taken the central premise of dating shows—that viewers can observe “ordinary” people in their search for love—but has coupled this with one of the most appealing aspects of Reality TV: the ability for the audience to shape content via active participation. Perhaps for this reason, *Love Island* has proven immensely popular, achieving consistently higher viewing figures and social media mentions than its rival programmes despite a relatively short run of just 8 weeks per year (Hallam, 2018; Waterson, 2018).

*Love Island's* producers have made little secret that generating audience engagement via social media has been a central aspect of their strategy. This approach seeks to elicit a feedback loop whereby television and social media content feed back onto each other in a cycle, driving audiences to engage with the show across multiple platforms (Lips, 2017). For *Love Island's* producers, enacting this strategy on a day-to-day basis has included: offering exclusive online content; using social media to post “first look” previews of upcoming television content; creating memes to share online via the show's official Twitter and Instagram accounts; utilizing polls and other games and quizzes online; sending notifications via the show's official app including 5 and 10 min pre-show alerts; making the app the only medium through which the audience may cast votes on the show; and creating a *Love Island* video game accessible through the app (Jones, 2018).

**Figure 1:**
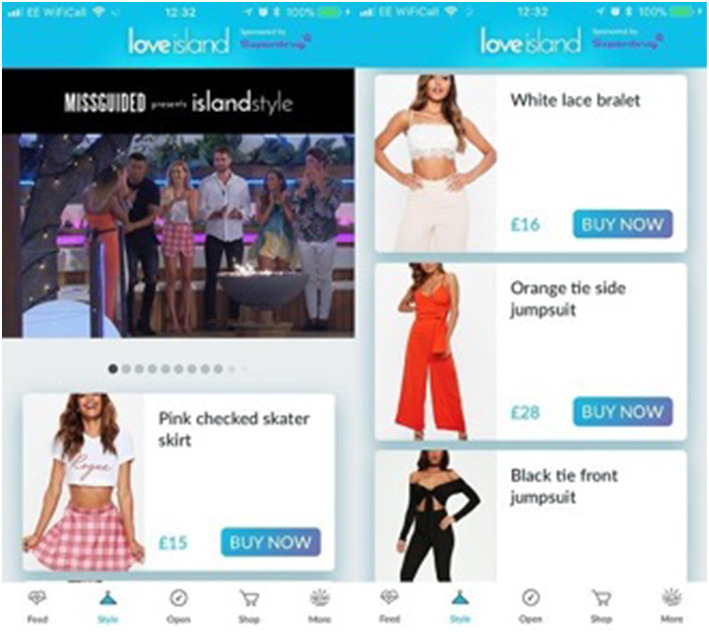
Screenshot of the “shop the look” feature on Love Island's app (Cole, 2018). Used with copyright holder's permission.

A key purpose of *Love Island's* strategy, and specifically the use of the app, is to act as a vehicle for the show's commercial interests. In its most recent series, *Love Island's* official commercial partners included Samsung, Superdrug, Rimmel London, Jet2Holidays, Missguided, Ministry of Sound, Kellogg's, Echo Falls, Primark, Lucozade Zero, and Thorpe Park (Scribe, 2018). It is perhaps the partnership with Missguided which best demonstrates *Love Island's* ability to push the boundaries of advertainment by using digital tools to exploit monetization opportunities. As part of its commercial partnership with *Love Island* in 2018, Missguided provided clothing for contestants to wear in the show. Audiences were then granted the opportunity to “shop the look” whereby they could buy the same outfits they saw contestants wearing. This process was mediated via the show's app which re-directed shoppers to Missguided's official website to complete their purchase (see Figure 1).

This innovative strategy has been variously described as the “future of shopping” (Faramarzi, 2018), a “marketing masterpiece” (Tuite, 2018), and a “multi-channel triumph… (and) one of the best TV partnerships ever (Cole, 2018). This and the rest of *Love Island's* commercial partnerships are designed to achieve more than simply consumer exploitation; they add another layer of interactivity in enabling audiences to undergo what might be understood as a wrap-around experience during their engagement with the show. Audiences can watch *Love Island* on television; discuss it online via social media; take part in polls and other games and quizzes with the official app; purchase official merchandise through the app or the show's official website; and even “shop the look” as described above. These interconnected services facilitate a short (insofar as engagement drops off once the show is over) but highly intense form of multi-platform engagement, maximizing audience participation, and by extension, potential for monetization (Gilliland, 2018).

But while these strategies offer new and innovative pathways toward audience engagement, the nature of participation in this context arguably remains limited to that dictated by the show's producers, such as casting votes and purchasing merchandise. In order to move beyond this structured and regulated form of participation, audiences have also engaged heavily across social media platforms such as Twitter, Snapchat, and Instagram. Twitter in particular appears to have emerged as the social media platform of choice for audience discussion and analysis. (Hallam, 2018) claims that in the week leading up to the finale of the show's fourth season in July 2018, Twitter accounted for over 81% of *Love Island* mentions across all major social media platforms. Further, at several instances during the summer of 2018, *Love Island*-related content appeared as the UK's top trending topic on Twitter, often outstripping the football World Cup. Indeed, *Love Island* was the most talked about television show in the UK on Twitter in 2018, with 6.3 million tweets and 2.5 billion Twitter impressions (Kantar Media, 2018), representing more than twice as many tweets as its nearest rival—quite a feat for a show which runs for only 8 weeks of the year.

An inevitable consequence of encouraging audience interactivity is that audiences will not only interact with the show but also with one another. Social media has enabled the *Love Island* audience to form what Rath (1985) once described as “invisible electronic networks,” demonstrated in the way fans of the show have congregated online and embraced social media as a platform for the development of a vibrant fan community. Rather than passively following *Love Island's* official Twitter account, audiences have shown signs of genuinely moving beyond traditional audience passivity through their engagement online. As well as interacting with one another using their own personal accounts, fans of the show have created bespoke, fan-led *Love Island* social media accounts which have enjoyed significant popularity. For instance, @*LoveIslandUK*, @*LIReactions*, and @*LoveIslandReact* have close to 70,000 Twitter followers between them while @*loveislandreactions* has over 414,000 Instagram followers^1^. Throughout *Love Island's* 8 week run in 2018, each account provided real-time commentary during and after nightly television broadcasts, creating memes, referring to ongoing fan community jokes, offering comedic reflections on the show's content and more generally interacting with other users. For the *Love Island* audience, consuming the show across numerous platforms has become not only completely normalized but also a central part of their enjoyment. Indeed, Manavis (2018) has proposed that “for an hour a day, Love Island made Twitter a kind place to be,” arguing that the show's friendly virtual community overcame the usually confrontational and toxic nature of social media. She claims that *Love Island* went so far as having “transformed the way we treat each other online” with discussions amongst *Love Island* fans being open and supportive. These reflections are supported by other users with comments at the end of the show's most recent run capturing such positive feelings:

“The actual best part of Love Island are the twitter conversations” (Amil, 2018)

“The best thing about #loveisland was twitter tbh… You made my evenings entertaining for the past 2 months and I thank you for that” (Dun, 2018)

“It has been such a p6gleasure connecting with people on Twitter over #LoveIsland… Thank you for making me smile, chortle, giggle & downright guffaw. For making me question things & for teaching me others” (Wozniak, 2018)

The cumulative result of both *Love Island's* deliberate multi-platform strategy as well as its audience's pro-active engagement online has therefore been the emergence of a tightly bound, highly connected and digitally-confident fan community pre-dominantly interacting via social media (Cavender, 2004). But the creation of an online community such as the *Love Island* audience may also lead to what Pierre Lévy (1997) once described as the creation of a “collective intelligence.” Writing in the late 1990s, Lévy predicted that the rising computerization of society would “promote the construction of intelligent communities in which our social and cognitive potential can be mutually developed and enhanced” (1997, p. 17). He proposed that as more and more people congregate online and interact with one another, information would be pooled and online communities would share and develop new forms of knowledge. Since “no one knows everything [but] everyone knows something” (1997, p. 13), communities would collectively become more intelligent thanks to the connective powers of the internet. As will be proposed later, this process can arguably be witnessed in the activities of the *Love Island* fan community.

## Participation, Interactivity, and Surveillance

Interactivity and participation run throughout contemporary surveillant relations (Lyon, 2018). While Orwellian notions of top-down surveillance carried out by powerful all-seeing actors are not completely obsolete, Lyon (2018) argues that these visions are dated. Instead, surveillance subjects are far less powerless and passive than Orwellian visions suggest and in fact, individuals and groups actively participate in so-called surveillance societies. They do so by offering up personal data every single day, habitually interacting with bodies which collect, process and share this personal data (Norris et al., 2017). Whether providing information concerning health records, employment experience, credit history, educational performance, or mundane everyday activities, it is commonplace for individuals to offer personal data to a vast range of public and private bodies. In the digital era, these practices have become so ubiquitous that it is virtually impossible to move through everyday life without engaging in multiple forms of participation with bodies composing the surveillant assemblage (Haggerty and Ericson, 2000). These habitual and pro-active forms of participation in surveillance practices need not necessarily be understood as inherently negative. Ball and Webster (2018) propose that different forms of participation may have vastly different outcomes and may be beneficial for some if, for instance, it makes them feel safer or offers them economic advantages.

The rapid growth of social media has not only facilitated a massive expansion of data collection practices by giants such as Facebook and Google, it has also created new opportunities for individuals to participate in the surveillance economy via modes of surveillance such as lateral surveillance and sousveillance (Mann et al., 2003; Andrejevic, 2004). Andrejevic (2004, p. 488) describes lateral surveillance as the function of “watching one another” and he explains that “lateral surveillance, or peer–to–peer monitoring (is) understood as the use of surveillance tools by individuals, rather than by agents of institutions public or private, to keep track of one another.” Social media has undoubtedly accelerated and vastly expanded the ability to carry out such activities (Mann and Ferenbok, 2013). Research has found that users of social media readily acknowledge that a key purpose of their engagement with platforms such as Facebook is to watch others (Joinson, 2008) and enact forms of “social surveillance” (Marwick, 2012). The concept of sousveillance, which was introduced by Mann et al. (2003), is also salient here. Sousveillance builds on (Mann, 1998) earlier notion of “reflectionism” as an example of individuals using technology to respond to surveillant power asymmetries. Sousveillance refers to bottom-up monitoring practices facilitated by the growth of affordable and accessible surveillance technologies such as mobile telephones and wearable computing devices. Mann et al. (2003, p. 331) describe sousveillance as a type of “inverse surveillance” essentially deployed “as a counter to organizational surveillance.” This ability to hold more powerful actors to account is the central *raison d'être* of sousveillance and represents an attempt by less powerful individuals to exercise greater agency and redress power imbalances at the heart of the panoptic asymmetries which characterize everyday life.

The increasing availability and affordability of mobile devices alongside the rapid expansion of social media has helped expand sousveillance practices in recent years. While past high-profile examples of sousveillance such as the filming of Rodney King's beating at the hands of the LAPD were once isolated albeit sensational, examples of police brutality and abuses of power by State or other actors are now captured and disseminated by members of the public almost every day. Social media grants individuals the ability to disseminate such content widely and instantaneously, by-passing traditional media, and in doing so, avoiding the filtering or regulatory mechanisms which traditional media remain subject to (Spiller and L'Hoiry, forthcoming). Thanks to social media, sousveillance practices have become so influential that they have seen the rise of seminal social movements such as Black Lives Matter, a movement largely mediated through social media and fuelled by recurrent instances of police brutality digitally captured by the public and disseminated online (Taylor, 2016). As such movements grow, sousveillance offers the potential for the formation of “communities of resistance” (Fernback, 2013) which group together online to monitor the actions of powerful actors.

The following section proposes that the *Love Island* fan community, driven by *Love Island's* producers' own strategy of pushing its audience to consume the show online as much as on television, has formed a community of resistance of sorts, in order to challenge the show's perceived lack of realism and authenticity.

## Love Island 2018, Sousveillance, and Challenging Claims of Realism

As outlined above, contemporary entertainment consumption, with Reality TV a prime example, is mediated across several platforms including television, social media, and mobile apps. *Love Island* presents a contemporary case study of these processes and the multiple indicators of success of the show—from viewing figures to online mentions^2^—suggest that the above-described audience engagement strategy has worked. However, the following section proposes that in pursuing this strategy, producers have given the *Love Island* audience both the means and the appetite to enact sousveillance practices holding the show's producers to account. The means have been provided via the drive to create an online fan community, pushed by *Love Island's* strategic, and heavy engagement with platforms such as Instagram, Twitter, and the show's mobile app—all forms of engagement which demand from the audience some basic level of digital competency in order to participate in crafting narratives in the show. The appetite comes in the form of the investment demanded of the audience which is implicit in their participation and engagement. As fans are encouraged to vote for their favorite contestants, to take part in quizzes, to discuss every major and minor controversy online, to use the show's official hashtag and to follow the show's official social media accounts, an inevitable sense of investment emerges for audiences. Of course, as discussed above, this investment can be commercially exploited, and *Love Island* has advanced to new heights the ways in which audience participation may be exponentially monetized. But with investment may come a sense of ownership for the *Love Island* fan community together with a collective responsibility to ensure that (mis)behaviors on the show are monitored and addressed. This is what appears to have taken place during *Love Island* 2018 and the following section offers a number of examples to demonstrate this.

The discussion below is based on a textual analysis of Twitter posts relating to *Love Island* in 2018. Both quantitative and qualitative analyses of social media content are well-established methodological approaches to explore public opinion and sentiment about a variety of topics (Thelwall et al., 2011; Marwick, 2013). Such methods take multiple forms (Pearce et al., 2018) and this paper deploys a qualitative approach. An initial manual observation of tweets concerning *Love Island* was carried out during the entire course of the show's run from 4 June, 2018 to 30 July, 2018 in order to observe behaviors of users discussing the show online. This initial observation noted repeated accusations of manipulation against *Love Island* producers and staff centered on key incidents and/or individuals during the show. As a result, a second more systematic analysis was undertaken focusing on these key incidents and individuals. At this point, the online software Mozdeh was deployed to capture all tweets using the hastag #loveisland together with key word combinations relating to the incidents and individuals in question (i.e., names of contestants). These searches were time-limited to the day of controversial incidents and the immediate 2 days following. The results of this search were then manually filtered to focus on users' discussions of the incidents and individuals in question. These tweets were manually analyzed and coded to explore the nature, tone, and sentiments of users' discussions. The primary data presented in this section is specifically selected to reflect the broader nature of the discussions relating to the incidents in question and to present the dominant feelings and sentiments among online discussions by *Love Island's* fan community.

### Example 1—#Kissgate

One of the show's most explosive moments came when New Jack^3^ and Georgia went on a date (Lewis, 2018). New Jack was at this time “coupled” with another contestant—Laura, seemingly one of Georgia's friends—but Georgia had nevertheless selected him for the date. Georgia was by this point well-known for relentlessly proclaiming her resolute loyalty to her friends. The date was therefore seen as a test of New Jack's relationship as well as Georgia's claims of loyalty. At the conclusion of the date, New Jack and Georgia appeared to kiss. But the controversy came in the fact that the camera angle made it difficult to determine whether the kiss had been mutual or whether Georgia had initiated the kiss before New Jack moved away. Upon their return to the villa, considerable, and heated discussions took place between contestants about the incident with both New Jack and Georgia proclaiming their respective innocence. In the aftermath of the show airing the date, footage of the kiss was analyzed relentlessly on social media by the *Love Island* audience with different supporters coming to different conclusions.

**Figure 2:**
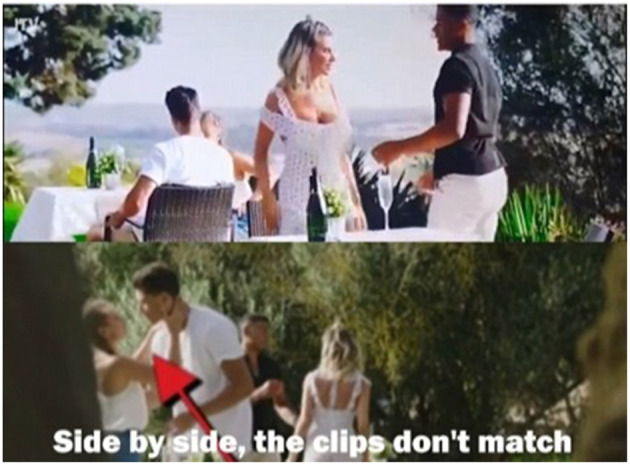
Screenshot of edited video footage showing the kiss was filmed twice (Shadijanova, 2018). Used with copyright holder's permission.

However, following several days of online debate about the veracity of New Jack and Georgia's actions, a user on Twitter released footage with accompanying analysis which appeared to show the incident from different camera angles. Rather than resolve the question of who initiated the kiss, the footage actually revealed that the kiss had been filmed on two separate occasions (see Figure 2).

Since the incident had originally stemmed from what appeared to be a single moment in which two people clumsily kissed, this revelation undermined the authenticity of the whole controversy. Viewers had been led to believe by *Love Island* producers that the kiss was a momentary incident, caught on camera from an angle which left questions unanswered and the contestants' intentions up for debate. In fact, the revelation that the footage had been filmed twice showed that the footage of the kiss had been carefully edited and curated to maximize the controversy and to fuel debate amongst the audience. This example demonstrates that without the investigatory work of *Love Island* viewers and their ability to generate previously undiscovered knowledge by drawing upon a “collective intelligence” (Lévy, 1997), this incident would have been presented as something which it fundamentally was not. At a broader level, this example adds to the ongoing debate concerning the constructed nature of Reality TV and whether this form of entertainment can truly claim to be authentic (Escoffery, 2006).

### Example 2—Samira and Frankie's Relationship

The way in which race is represented in Reality TV has been a long-running area of concern (Orbe, 2008; Squires, 2008). *Love Island* has appeared to continue this uneasy trend, with some commentators reflecting that non-white female contestants have rarely fared well on the show (Adegoke, 2018) and that black female contestants in Reality TV more broadly are often narrowly presented as the “angry black woman” (Dash, 2018). In *Love Island* 2018, the show's only black female contestant Samira appeared to be cast very quickly as being “unlucky in love.” This narrative began as soon as she was not picked by male contestants to be in a couple during the show's opening episode and instead had to enter into a platonic and often awkward relationship with another contestant.

After several weeks, Samira's luck seemed to change with the arrival of new contestant Frankie. Samira and Frankie began a romantic relationship, but this lasted little more than 2 weeks before Frankie was voted out of the island. Frankie's removal prompted an emotional reaction from Samira who lamented losing him when their relationship was blossoming (Gibb, 2018). This reaction led to considerable discussion amongst the *Love Island* audience online, some of whom seemed confused by the apparent depth of Samira's investment in the relationship when relatively little screen-time had been dedicated to showing what Samira appeared to believe was a promising romance.

“Anyone watching love island for the first time would think Frankie had just died not left after a week of knowing Samira” (Bumby, 2018)

“Not being funny but Samira crying unctrollably like someone dropped her best friend into a piranha tank.” (Ebuwa, 2018)

Before long, accusations began to surface that *Love Island's* producers had deliberately limited coverage of Samira and Frankie's relationship in order to maintain Samira's “unlucky in love” narrative. These allegations were further fuelled when it was revealed that Samira and Frankie had spent a night the villa's hideaway—an action considered a big step forward in couples' relationships on the show—but this was edited out and never broadcast despite the fact that such encounters are routinely televised (Saunders, 2018). The *Love Island* fan community reacted furiously online, accusing producers of deliberate attempts to undermine Samira's relationship and even of racial pre-judice:

“How can you justify editing out 99% of Samira and Frankie, including their hideaway stay. Careful #loveisland your pre-judice is showing” (Wilkinson, 2018)

“samira and frankie went to the HIDEAWAY!! where was that!??! the producers did that girl DIRTY from the start and if you refuse to see that, check urself” (Lauren, 2018)

“Nahh, Samira and Frankie had a night in the hideaway and it wasn't shown?? We didn't see 99% of their relationship and winning Love Island is RELIANT on public popularity. The producers made a conscious decision to sabotage them smh” (Avocaldo, 2018).

Shortly thereafter, Samira left the show of her own volition. In direct response to this perceived imbalance in the presentation of Samira and Frankie's relationship on the show, *Love Island* fans began to disseminate examples of the couple's relationship back in the UK, re-tweeting and liking photos and videos showing the couple enjoying the early stages of their romance. This collective reaction demonstrates the ability of the *Love Island* fan community to firstly call attention to the alleged misrepresentation of a contestant's narrative but also to then re-shape this narrative toward one which is more aligned to the audience's preferences. Based on a suspicion that they had been shown only a selective account of the couple's relations in the show, fans deployed counter measures to challenge the narrative ascribed to Samira by *Love Island's* producers and instead celebrated her relationship with Frankie whilst concurrently calling attention to the producers' apparent decision to reduce exposure of this romance. What is also perhaps demonstrated here is the depth of distrust amongst Reality TV audiences. Rather than this example representing only viewers' grievances concerning a lack of screen time for one of their favorite contestants, the accusations made in the tweets above demonstrate that for some fans, Samira's narrative was one rooted in racial pre-judice amongst *Love Island's* producers. The severity of such accusations hints at a deep-seated distrust arguably grounded in the long-term legacy of producer subterfuge in Reality TV.

### Example 3—Jack in Casa Amore and Dani's Reaction

A recurring dramatic device in *Love Island* is the moment when couples are split and male contestants spend a week in a different villa—Casa Amore—where they meet a host of new female contestants. Meanwhile, the female contestants in the main villa also meet new male contestants. The premise behind this exercise is to test the strength of existing relationships and the week culminates in a dramatic ceremony during which contestants decide whether to return to their previous couples or form new relationships. In *Love Island* 2018, Jack and Dani were consistently seen as having the longest-running and strongest relationship in the villa and were firm fan favorites. However, when Jack arrived at Casa Amore, his (non-*Love Island*) ex-girlfriend Ellie was waiting, prompting an anxious reaction from Jack who was evidently uneasy about her appearance. Jack made it clear on several instances that his concern was that Dani would be worried about Ellie's presence. He went so far as to sleep outside throughout the week in order to make clear that he did not want to spend time with or sleep in the same room as Ellie (Taylor, 2018). However, later in the week, *Love Island's* producers showed Dani footage of Jack's behavior in Casa Amore. Omitted from this footage was his decision to sleep outside and instead, only his initial, anxious reaction was shown with little additional context, leading Dani to become visibly distressed. Dani's upset at what fans judged to be an out-of-context clip resulted in outcry online and accusations that producers had engaged in emotional manipulation to maximize the incident's dramatic effect:

“What you're doing to this girl is psychological warfare Love Island… you're misleading her about Jack. That's dirty and cruel. #loveisland #mentaltorture #unfair” (CheyenneMonty, 2018)

“Out of order. switched off for the rest of the series. you think it makes good tv but that's someone's emotions” (Allison, 2018)

“I was really enjoying @LoveIsland like I forgot how toxic manipulative reality tv was, but that Dani stunt was so twisted I don't know if I can continue to watch a show that encourages the deterioration of someone's mental health. Love Island wants people to be mentally unwell.” (Donnell, 2018)

Importantly, this discontent went beyond fans simply complaining to one another online. Instead, they mobilized and took proactive steps by submitting formal complaints to the UK's regulatory authority for broadcasting, the Office of Communications–better known as Ofcom. Over 2,500 complaints were filed to Ofcom for this incident alone, representing one of the most complained about single incidents on television in 2018^4^. Indeed, despite a relatively short run of 8 weeks, *Love Island* attracted the fourth highest number of complaints in 2018^5^ (Ofcom, 2018). The behavior of the *Love Island* audience in this example, and indeed in the numerous other instances in which complaints were made to Ofcom during *Love Island's* 2018 run, demonstrates how collective outrage about perceived misbehavior by the show's producers can go beyond passive audience dissatisfaction and, fuelled by the collective power generated by being members of a community of resistance, fans can and will proactively hold producers to account. Comparing the numbers of Ofcom complaints in 2018 with that of previous *Love Island* series is also illustrative. In 2016, the show received only 40 complaints while the 2017 edition received 135 (Corrodus, 2018). The considerable rise in such complaints in 2017 may therefore suggests not only an increase in viewership but also the mounting sense of agency amongst *Love Island's* audience amidst the growing collectivism of the show's fan community.

### Example 4—Questioning the Missing Challenges

A key feature of *Love Island* is the series of challenges contestants must complete throughout the show's run. These challenges often feature as the highlights of each series and are eagerly anticipated by the *Love Island* audience. However, with the 2018 series nearing its conclusion, *Love Island* fans began to vent their frustrations online that some of the best-loved challenges had not taken place:

“where's the parents? the lie detector? the babies? the guess who said that about ya game? give us more challenges i'm boredddd of them sat around” (Cousins, 2018)

“The whole of the UK waiting for the lie detector test, baby challenge and meet the parents to happen” (Jasmin, 2018)

“Honestly it's so close to the end…why bring new ppl…let's do the call home, then lie detector, give them the babies, then meet the parents and other challenges…we don't need no more new ppl at this point” (Mrsd, 2018)

Whether by design or otherwise, in *Love Island* 2018, the final week of the show featured all the challenges fans had been demanding online. In previous series, challenges usually took place around once a week, so such a concentration of the show's most popular challenges in the final week of the 2018 series certainly appeared highly unusual. There is a suggestion here that *Love Island's* producers responded directly to fans' dissatisfaction at the absence of these challenges. If this is true, it proposes a fascinating example of the way in which audiences can shape the activities contestants take part in seemingly through sheer force of will. Rather than contributing to the show's content within parameters defined by producers (i.e., taking part in scheduled votes with only certain contestants to choose from), fans are breaking beyond these constraints and making their own demands. The *Love Island* fan community's collective power harnessed online appears to be forcing producers into producing content on demand, hinting at a broader shift in the power relations between producers and consumers.

Whether the examples above can truly be said to demonstrate sousveillant practices may be up for debate. *Love Island's* fans are certainly not seeking to counter organizational surveillance in order to destroy these systems. Indeed, it seems clear that despite their complaints, *Love Island's* fans do not want the show to fail, as demonstrated by the strong viewing figures for the show's finale which suggest that audiences keep watching even after uncovering staged incidents earlier in the series. *Love Island* fans do however demonstrate motivations closely aligned to sousveillant activities insofar as they are attempting to redress asymmetries of power, specifically the power of producers to engineer incidents to maximize dramatic content and the audience's previous inability to directly challenge such content. The example of #kissgate is aligned to traditional sousveillance methods in the sense that new footage was generated to counter a dominant narrative. The other examples use tactics perhaps not usually associated with sousveillant practices, but all are designed to challenge producers when their behaviors stray from the collective values and ideals of the *Love Island* fan community.

At the heart of these examples is a search for authenticity among *Love Island's* fans, reflecting a continuing longing for realism amongst Reality TV audiences whose trust in this genre has been broken by a legacy of duplicitous behavior (Heritage, 2019). When events appear staged or producers are suspected of skewing the veracity of the content presented to the audience, fans enact their collective power to challenge these false narratives. The question is however, whether this truly represents a problem for *Love Island's* producers. Whether criticizing or praising producers, *Love Island* audiences discussing the show online will still use the *Love Island* hashtag and that alone may be enough to count as a success for *Love Island's* producers and their sponsors. The old adage that there's no such thing as bad publicity may be true, and the record-breaking commercial partnerships already agreed ahead of the 2019 edition of the show (Sweeney, 2019) suggest *Love Island's* producers have little to be concerned about—in the short term at least.

In the longer term however and as the examples of perceived subterfuge by producers outlined above add up, they become part of a longer history of deception in Reality TV and continue to chip away at any residual belief that Reality TV offers genuine authenticity and realism. For the audience, ongoing accusations that outcomes are pre-determined or fixed, and that controversial incidents are faked and stage-managed may, over time, undermine the attraction of shows like *Love Island*. But perhaps more than anything, what the actions of the *Love Island* fan community reveal is an enduring tension for all Reality TV audiences: between a desire for authenticity and an acceptance (or an acquiescence to) the fact that Reality TV is ultimately staged to some extent. *Love Island* fans enjoy the show and want to keep watching—hence despite their frustration at various duplicitous behaviors by producers, viewing figures remain high even after accusations of racism, emotional manipulation, and staged controversies (Waterson, 2018). Critically however, rather than switching off altogether, *Love Island* fans instead seek to consume the show on their *own terms*, binding together to re-craft narratives when the content presented to them by producers does not meet with the audience's approval.

## Conclusion

As television producers continue to push for consumption of their products across multiple platforms, audiences are finding new ways to consume and engage with popular entertainment such as Reality TV. The potential for commercial exploitation and monetization of audience participation in this context is limitless and it is this potential which is driving television producers and their sponsors to encourage audiences to move beyond passive consumption of fixed content. Instead, they seek to co-opt their audiences as co-producers who discuss, analyze, and commercially engage with a show before, during, and after its live broadcast. While the commercial rewards are significant and growing, these developments inevitably push audiences toward greater investment in these shows and a vested interest in monitoring outcomes relative to the audience's own desires.

These developments have correlated with the growth of social media in the past 15 years which has brought with it a greater potential for individual power and agency thanks to the communicative and connective capacity of different social media platforms. The ability of individuals to more easily and instantaneously connect online has engendered a sense of collective power, particularly when these individuals can identify and group together around a shared interest such as fandom of a television show. As Cover explains:

A digital environment promoting interactivity has fostered a greater capacity and a greater interest by audiences to change, alter and manipulate a text or a textual narrative, to seek co-participation in authorship, and to thus redefine the traditional author-text-audience relationship (Cover, 2006, p. 140).

As online communities come together, discuss shared interests and pool their knowledge, a “collective intelligence” (Lévy, 1997) begins to emerge which may be used to monitor the behaviors of others. The continuing rise of lateral surveillance and sousveillance practices demonstrates how this “collective intelligence” can be re-purposed within the work of virtual “communities of resistance” (Fernback, 2013). The behavior of *Love Island's* fans in 2018 demonstrates these practices in the context of Reality TV. Fans of the show mobilized online and on multiple occasions took pro-active steps, emboldened by the collective spirit of these online communities, to hold *Love Island's* producers to account for perceived misbehaviors and the undercutting of the show's claims to authenticity. The question remains what the impact of such audience behaviors is—do these behaviors in fact offer *Love Island* greater exposure and is therefore welcomed by its producers regardless of whether fan reactions are positive or critical? Or, by relentlessly driving their audience toward online consumption of the show, have *Love Island's* producers created a monster whose behaviors they can no longer predict or control? In an age of post-truth politics, “alternative facts” and “fake news,” if fans continue to feel duped as they reveal instances of staged controversies or deliberate manipulation of contestants, might they become so disenchanted by a lack of authenticity and realism that, in the end, they switch the channel?

## Data Availability

The raw data supporting the conclusions of this manuscript will be made available by the authors, without undue reservation, to any qualified researcher.

## Author Contributions

The author confirms being the sole contributor of this work and has approved it for publication.

### Conflict of Interest Statement

The author declares that the research was conducted in the absence of any commercial or financial relationships that could be construed as a potential conflict of interest.

## Footnotes

^1^The official *Love Island* Twitter and Instagram accounts have 989,000 and 2.5 million followers, respectively. As such, although the fan-led accounts discussed above are not comparable to official accounts purely in terms of follower numbers, they nevertheless arguably boast high numbers of followers for non-official, fan-led accounts.

^2^*Love Island* attracted more viewers in 2018 than any of its previous series with ~4 million viewers watching the live finale (Waterson, 2018). The 2018 series also generated 2.5 billion twitter impressions, outstripping the 2017 series which reached 1.5 billion impression (Lips, 2017).

^3^New Jack was so-called in order to differentiate him from another contestant called Jack who had been on the show from the first episode.

^4^As an aside, it is worth noting that in dismissing these complaints, Ofcom drew attention to the fact that in Reality TV shows such as *Love Island*, viewers should expect scenes to be “engineered to test contestants' relationships” (Sporn, 2018). This response alludes to Ofcom's recognition that *Love Island's* content is constructed or scripted in nature.

^5^Incidentally, 540 complaints made to Ofcom related to perceived unfair editing of Samira on the show, relating to Example 2 above (Ofcom, 2018).

## References

[B1] Adegoke Y. (2018, 6 26). Single black female: Love Island and the problem with race and dating. The Guardian. Available online at: https://www.theguardian.com/lifeandstyle/shortcuts/2018/jun/26/single-black-female-love-island-the-problem-with-race-and-dating (accessed March 11, 2019).

[B2] Allen K. Mendick H. (2013). Keeping it Real? Social Class, Young People and ‘Authenticity' in Reality TV. Sociology 47, 460–476. 10.1177/0038038512448563

[B3] Allison H. (2018, 7 1). Available online at: https://twitter.com/HayleyJane32/status/1013528126436139008 (accessed March 12, 2019).

[B4] Amil (2018, 7 26). Available online at: https://twitter.com/amil/status/1022427558774820864 (accessed March 12, 2019).

[B5] Anderson K. (1978). Television Fraud: The History and Implications of the Quiz Show Scandals. Westport, CT: Greenwood Press.

[B6] Andrejevic M. (2004). The work of watching one another: lateral surveillance, risk, and governance. Surveill. Soc. 2, 479–497. 10.24908/ss.v2i4.3359

[B7] Andrejevic M. (2008). Watching television without pity: the productivity of online fans. Television New Media 9, 24–46. 10.1177/1527476407307241

[B8] Avocaldo (2018, July 12). Available online at: https://twitter.com/avocadlo/status/1017472760082259969 (accessed June 20, 2019).

[B9] Ball K. Webster W. (2018). Surveillance and Democracy in Europe. Oxon: Routledge.

[B10] Biressi A. Nunn H. (2005). Reality TV: Realism and Revelation. London: Wallflower Press.

[B11] Bumby (2018, 7 11). Available online at: https://twitter.com/Aaron_Bumby/status/1017162882415517698 (accessed June 20, 2019).

[B12] Campelli M. (2015, 3 9). First Dates pulls best ever audience. Broadcast. Available online at: https://www.broadcastnow.co.uk/first-dates-pulls-best-ever-audience/5084024.article (accessed June 20, 2019).

[B13] Cavender G. (2004). In search of community on reality TV, in Understanding Reality Television, eds Holmes S. Jermyn D. (Oxon: Routledge), 154–172.

[B14] CheyenneMonty (2018, 7 1). Available online at: https://twitter.com/CheyenneMonty/status/1013528434079928322 (accessed June 20, 2019).

[B15] Cole S. (2018, 7 30). Why the Love Island & Missguided partnership was a multichannel triumph. Econsultancy. Available online at https://econsultancy.com/why-the-love-island-missguided-partnership-was-a-multichannel-triumph/ (accessed March 11, 2019).

[B16] Corrodus C. (2018, 8 3). Love Island sparks more than 4,000 complaints. The Telegraph. Available online at: https://www.telegraph.co.uk/tv/2018/08/03/love-island-sparks-4000-ofcom-complaints/ (accessed June 20, 2019).

[B17] Cousins A. (2018, 7 18). Available online at: https://twitter.com/adelecousins/status/1019686318996148225 (accessed March 12, 2019).

[B18] Cover R. (2006). Audience inter/active: Interactive media, narrative control and reconceiving audience history. New Media Soc. 8, 139–158. 10.1177/1461444806059922

[B19] Dash D. (2018, 8 28). Why is Reality TV so obsessed with the ‘angry black woman'? The Guardian. Available online at: https://www.theguardian.com/culture/2018/aug/28/why-is-reality-tv-so-obsessed-with-the-angry-black-woman (accessed May 20, 2019).

[B20] Deans J. (2007, 9 26). GMTV phone-in scandal: the biggest fraud in UK TV history? The Guardian. Available online at: https://www.theguardian.com/media/organgrinder/2007/sep/26/gmtvphoneinscandalthebigge (accessed June 20, 2019).

[B21] Deery J. (2004). Reality TV as advertainment. Popular Commun. 2, 1–20. 10.1207/s15405710pc0201_1

[B22] Donnell A. O. (2018, 7 1). Available online at https://twitter.com/AineODonnellx/status/1013631838429081600 (accessed March 11, 2019).

[B23] Doyle A. (1998). ‘Cops': television policing and policing reality, in Entertaining Crime: Television Reality Programs, eds Fishman M. Cavander G. (New York, NY: Aldine de Gruyter), 95–114.

[B24] Dun (2018, 7 31). Available online at: https://twitter.com/Dun_03/status/1024384198331781120 (accessed June 20, 2019).

[B25] Ebuwa (2018, 7 10). Available online at: https://twitter.com/VicEbuwaSlick/status/1016775648428412931 (accessed June 21, 2019).

[B26] Escoffery D. S. (ed) (2006). How Real is Reality Television? Essays on Representation and Truth. Jefferson, NC: McFarland & Company Inc.

[B27] Faramarzi S. (2018, 6 6). Love at first swipe: would you shop the Love Island look live? The Guardian. Available online at: https://www.theguardian.com/fashion/2018/jun/06/love-at-first-swipe-would-you-shop-the-love-island-look-live (accessed March 11, 2019).

[B28] Fernback J. (2013). Sousveillance: Communities of resistance to the surveillance environment. Telem Inform. 30, 11–21. 10.1016/j.tele.2012.03.003

[B29] Fetveit A. (1999). Reality TV in the digital era: a paradox in visual culture? Media Culture Soc. 21, 787–804. 10.1177/016344399021006005

[B30] Gibb J. (2018, 7 12). Love Island's Samira Might leaves the villa after devastating heartbreak over Frankie Foster. The Mirror. Available online at: https://www.mirror.co.uk/tv/tv-news/breaking-love-islands-samira-mighty-12904565 (accessed July 15, 2019).

[B31] Gilder G. (1994). Life After Television: The Coming Transformation of Media and American Life. New York, NY: W. W. Norton and Company.

[B32] Gillan J. (2004). From Ozzie Nelson to Ozzy Osbourne: The genesis and development of the reality (star) sitcom, in Understanding Reality Television, eds Holmes S. Jermyn D. (London: Routledge), 54–70.

[B33] Gilliland N. (2018, 6 20). Five social media lessons marketers can learn from Love Island. Econsultancy. Available online at: https://econsultancy.com/five-social-media-lessons-marketers-can-learn-from-love-island/(accessed March 11, 2019).

[B34] Haggerty K. D. Ericson R. V. (2000). The Surveillant Assemblage. Br. J. Sociol. 51, 605–622. 10.1080/00071310020015280 11140886

[B35] Hall A. E. (2009). Perceptions of media realism and reality TV, in The Sage Handbook of Processes and Effects, eds Nabi R. L. Oliver M. B. (Thousand Oaks: Sage), 423–438.

[B36] Hallam (2018, 8 2) How Love Island is shaping digital-first television. Hallam. Available online at: https://www.hallaminternet.com/love-island-social-media-strategy/ (accessed March 11, 2019).

[B37] Heritage S. (2019, 10 25). From Bake Off to Strictly: why do so many believe that reality TV is fixed? The Guardian. Available online at: https://www.theguardian.com/tv-and-radio/2018/oct/25/great-british-bake-off-strictly-come-dancing-x-factor-why-do-people-believe-that-reality-tv-is-fixed (accessed July 23, 2019).

[B38] Hill A. (2002). Big brother: the real audience. Television New Media 3, 323–40. 10.1177/152747640200300307

[B39] Hill A. (2014). Reality TV. Oxon: Routledge.

[B40] Holmes S. (2004). ‘But This Time You Choose!' Approaching the ‘Interactive' Audience in Reality TV. Int J Cult Stud. 7, 213–231. 10.1177/1367877904043238

[B41] Jasmin (2018, 7 19). Available online at: https://twitter.com/ItsJasminHere/status/1020044355372011521 (accessed March 12, 2019).

[B42] Jenkins H. (2006). Convergence Culture: Where Old and New Media Collide. New York, NY: NYU Press.

[B43] Joinson A. N. (2008). 'Looking at', ‘Looking up' or ‘Keeping up with' People? Motives and Uses of Facebook, in CHI 2008 Proceeding, April 5–10, 2008 (Florence), 1027–1036.

[B44] Jones J. M. (2003). Show your real face: A fan study of the UK *Big Brother* transmissions (2000, 2001, 2002)–investigating the boundaries between notions of consumers and producers of factual television. New Media Soc. 5, 400–421. 10.1177/14614448030053006

[B45] Jones W. (2018) The Love Island video game is insane–and also sort of worth playing. Her. Available online at: https://www.her.ie/entertainment/love-island-video-game-insane-also-sort-worth-playing-411952 (accessed March 11, 2019).

[B46] Kantar Media (2018, 12 11). Love Island Beats Politics When it Comes to Most Engaging Television Content. Available online at: https://www.kantarmedia.com/uk/newsroom/press-releases/love-island-beats-politics-when-it-comes-to-most-engaging-television-content (accessed March 11, 2019).

[B47] Kjus Y. (2009). Idolizing and monetizing the public: the production of celebrities and fans, representatives and citizens in reality TV. Int. J. Commun. 3, 277–300.

[B48] Lauren (2018, 7 12). Available online at: https://twitter.com/LaurenLouise_Mc/status/1017503239644774401 (accessed June 21, 2019).

[B49] Lévy P. (1997). Collective Intelligence: Mankind's Emerging World in Cyberspace. Transl. by R. Bononno. New York, NY: Plenum Press.

[B50] Lewis A. (2018, 7 9). Love Island's Georgia and Jack kiss in slow-mo in case you still aren't sure what happened. Cosmopolitan. Available online at: https://www.cosmopolitan.com/uk/entertainment/a22086231/love-island-georgia-jack-kiss-video/ (accessed July 15, 2019).

[B51] Lips A. (2017, 9 22).“Love Island: How ITV used social media to drive sales and viewers to hit their hit series. Social Media Week. Available online at: https://socialmediaweek.org/blog/2017/09/love-island-itv-used-social-drive-viewing-sales-hit-series/ (accessed March 11, 2019).

[B52] Lyon D. (2018). Culture of Surveillance: Watching as a Way of Life. Cambridge: Polity Press.

[B53] Manavis S. (2018, 8 3). For an hour a day, Love Island made Twitter a nice place to be. NewStatesman. Available online at: https://www.newstatesman.com/culture/tv-radio/2018/08/hour-day-love-island-made-twitter-kind-place-be (accessed March 11, 2019).

[B54] Mann S. (1998). 'Reflectionism' and 'diffusionism': new tactics for deconstructing the video surveillance superhighway. Leonardo 31, 93–102. 10.2307/1576511

[B55] Mann S. Ferenbok J. (2013). New Media and the power politics of sousveillance in a surveillance-dominated world. Surveil. Soc. 11, 18–34. 10.24908/ss.v11i1/2.4456

[B56] Mann S. Nolan J. Wellman B. (2003). Sousveillance: inventing and using wearable computing devices for data collecting in surveillance environments. Surveil. Soc. 1, 331–355. 10.24908/ss.v1i3.3344

[B57] Marwick A. E. (2012). The public domain: social surveillance in everyday life. Surveil. Soc. 9, 378–393. 10.24908/ss.v9i4.4342

[B58] Marwick A. E. (2013). Ethnographic and qualitative research on Twitter in Twitter and Society, eds Weller K. Bruns A. Puschmann C. Burgess J. Mahrt M. (New York, NY: Peter Lang), 109–122.

[B59] Mrsd (2018, 7 15). Available online at: https://twitter.com/RealMrsD_/status/1018642464469643264 (accessed June 21, 2019).

[B60] Nabi R. (2007). Determining dimensions of reality: a concept mapping of the reality TV landscape. J. Broadcast. Electr. Media 51, 371–390. 10.1080/08838150701307111

[B61] Negroponte N. (1995). Being Digital. New York: Alfred A. Knopf.

[B62] Norris C. De Hert P. L'Hoiry X. Galetta A. (eds.). (2017). The Unaccountable State of Surveillance. Springer.

[B63] Ofcom (2018, 12 27) Most complained about TV programmes of 2018. Available online at: https://www.ofcom.org.uk/about-ofcom/latest/media/media-releases/2018/most-complained-about-tv-programmes-of-2018 (accessed March 11, 2019).

[B64] Orbe M. P. (2008). Representations of race on reality TV: watch and discuss. Crit Stud Media Commun. 25, 354–352. 10.1080/15295030802327790

[B65] O'Reilly T. (2005). What is Web 2.0? Available online at: http://www.oreillynet.com/pub/a/oreilly/tim/news/2005/09/30/what-is-web-20.html (accessed June 20, 2019).

[B66] Papacharissi Z. Mendelson A. L. (2007). An Exploratory Study of Reality Appeal: Uses and Gratifications of Reality TV Shows. J. Broadcast. Electr. Media 51, 355–370. 10.1080/08838150701307152

[B67] Pearce W. Özkula S. M. Greene A. K. Teeling L. Bansard J. S. Janna Joceli Omena J. J.. (2018). Visual cross-platform analysis: digital methods to research social media images. Inform. Commun. Soc. 10.1080/1369118X.2018.1486871

[B68] Rath C. D. (1985). The invisible network: television as an institution in everyday life, in Television in Transition: Papers from the First International Television Studies Conference, eds P. Drummond and R. Patterson (London: Film Studies Institute).

[B69] Razaghpanah A. Nithyanand R. Vallina-Rodriguez N. Sundaresan S. Allman M. Kreibich C.. (2018). Apps, Trackers, Privacy, and Regulators A Global Study of the Mobile Tracking Ecosystem. Available online at https://haystack.mobi/papers/ndss18_ats.pdf (accessed March 11, 2019).

[B70] Saunders E. (2018, 7 12). Samira and Frankie's secret Hideaway night of passion has been edited out of show. The Mirror. Available online at: https://www.mirror.co.uk/tv/tv-news/love-island-samira-frankie-hideaway-12905152 (accessed March 18, 2019).

[B71] Scribe (2018). Inside the Villa: How Love Island Made a Sizzling Success of Social-First TV, Social Chain. Available online at: https://www.socialchain.com/scribe/inside-the-villa-how-love-island-made-a-sizzling-success-of-social-first-tv/ (accessed July 23, 2019).

[B72] Shadijanova D. (2018, 7 10). These two videos prove that Love Island is actually staged. The Tab. Available online at: https://thetab.com/uk/2018/07/10/love-island-georgia-jack-kiss-staged-72203 (accessed March 18, 2019).

[B73] Spiller K. L'Hoiry X. (forthcoming). Watch Groups, Surveillance Doing it for Themselves. Surveillance & Society.

[B74] Sporn N. (2018, 7 16). Love Island will not be investigated by Ofcom despite 2,600 complaints over Dani Dyer clip. Evening Standard. Available online at: https://www.standard.co.uk/showbiz/celebrity-news/love-island-will-not-be-investigated-by-ofcom-despite-2600-complaints-over-dani-dyer-clip-a3888541.html (accessed June 20, 2019).

[B75] Squires C. (2008). Race and reality TV: tryin' to make it real–but real compared to what? Crit. Stud. Media Consump. 25, 434–440. 10.1080/15295030802328038

[B76] Sweeney M. (2019, 1 31). ITV's Love Island lands record sponsorship with Uber Eats. The Guardian. Available online at: https://www.theguardian.com/business/2019/jan/31/itv-love-island-lands-record-sponsorship-deal-with-uber-eats (accessed March 11, 2019).

[B77] Taylor F. (2018, 7 2). The Love Island producers have made a big error of judgement over Dani and Jack. Radio Times. Available online at: https://www.radiotimes.com/news/tv/2018-07-02/love-island-2018-producers-jack-dani-error-over-casa-amor-itv2/ (accessed July 15, 2019).

[B78] Taylor K. (2016). From #blacklivesmatter to Black Liberation. Chicago, IL: Haymarket Books.

[B79] Thelwall M. Buckley K. Paltoglou G. (2011). Sentiment in Twitter events. J. Am. Soc. Informn. Sci. Technol. 62, 406–418. 10.1002/asi.21462

[B80] Tuite H. (2018, 7 30). Love Island–a marketing masterpiece. *Tuna Fish Media*. Available online at: https://tunafishmedia.co.uk/love-island-a-marketing-masterpiece/ (accessed March 11, 2019).

[B81] Waterson J. (2018, 7 31). Love Island final attracts more than 4 million viewers. The Guardian. Available online at: https://www.theguardian.com/tv-and-radio/2018/jul/31/love-island-final-attracts-more-than-4-million-viewers-dani-dyer-jack-fincham (accessed June 20, 2019).

[B82] Wilkinson C. (2018, 7 12). Available online at: https://twitter.com/caitrosewilk/status/1017430670446977026 (accessed March 12, 2019).

[B83] Woodward E. (2018, 8 20). 8 Times ‘Keeping up with the Kardashians' Was Accused of Being Fake. Buzzfeed. Available online at: https://www.buzzfeed.com/elliewoodward/keeping-up-with-the-kardashians-fake-allegations (accessed June 20, 2019).

[B84] Wozniak G. (2018, 7 30). Available online at: https://twitter.com/glyniswozniak/status/1024078211704139776 (accessed March 12, 2019).

